# Enteropathogenic *E. coli* effector Map interacts with Rab13 and regulates the depletion of the tight junction proteins occludin and claudins via cathepsin B-mediated mechanisms

**DOI:** 10.1242/bio.061794

**Published:** 2025-02-27

**Authors:** Anupam Mandal, Pangertoshi Walling, Shirin Qureshi, Kritika Kansal, Saima Aijaz

**Affiliations:** Special Centre for Molecular Medicine, Jawaharlal Nehru University, New Delhi 110067, India

**Keywords:** Tight junctions, Intestinal barrier, Enteropathogenic *Escherichia coli*, Map, Cathepsins, Rab13

## Abstract

Infections by enteropathogenic *Escherichia coli* (EPEC) cause acute diarrheal disease in infants accounting for severe morbidity and mortality. One of the underlying causes of the disease is the breakdown of the intestinal barrier maintained by the tight junctions (TJs). EPEC uses a type 3 secretion system to translocate more than 20 effectors into infected cells, which disrupt several functions of the host cells. The effectors EspF, Map, EspG1/G2 and NleA have been reported to disrupt the TJs causing the leakage of charged ions and uncharged molecules through the barrier. We have previously reported that EspF and Map cause the depletion of TJ proteins claudin-1, claudin-4 and occludin through both transcriptional and post-transcriptional mechanisms. Here, we show that the inhibition of the lysosomal protease cathepsin B, in cells expressing the EPEC effector Map, reduces the depletion of claudin-1, claudin-4 and occludin. Further, we show that the expression of a mutant Map protein lacking the mitochondrial targeting sequence inhibits the depletion of occludin and its delocalization from the TJs and partially rescues claudin-4 levels and its junctional localization. We also identified a novel interaction of Map with the GTPase Rab13. Rab13 has been reported to mediate the recycling of occludin to the plasma membrane. Since occludin regulates the passage of macromolecules through the intestinal TJ barrier, the interaction of Map with Rab13 may have important implications for the loss of TJ integrity and excessive leakage through the intestinal barrier in EPEC pathogenesis.

## Teaser

**Summary:** Enteropathogenic *E. coli* infection causes acute diarrheal disease in infants. This paper shows that the EPEC effector Map interacts with the recycling endosome marker Rab13 and disrupts the intestinal barrier by cathepsin B-mediated depletion of tight junction proteins.

## INTRODUCTION

Enteropathogenic *Escherichia coli* (EPEC) infection results in the rapid onset of diarrhea in infants in the developing world, causing significant morbidity and mortality (Hodges and Gill, 2010; Nataro and Kaper, 1998; Ochoa and Contreras, 2011). The infection is characterized by the disruption of the intestinal tight junction (TJ) barrier, defects in the sodium-glucose co-transporter 1 (SGLT-1), Na^+^/H^+^ exchanger isoform 3 (NHE3), Cl^−^/HCO_3_^−^ exchanger (also known as downregulated in adenoma, DRA) and aquaporins (Croxen and Finlay, 2010; Guttman and Finlay, 2008; Viswanathan et al., 2009). The combined effect of these disruptions results in the excessive leakage of solutes and ions through the intestinal layer as well as reduced absorption of nutrients by the enterocytes (Vallance and Finlay, 2000; Viswanathan et al., 2009). EPEC uses a type 3 secretion system (T3SS) to translocate at least 20 effector proteins into the infected enterocytes (Dean and Kenny, 2009; Dean et al., 2005; Wong et al., 2011). Once inside the host cell, these effector proteins target multiple cellular organelles and disrupt their functions. Of the more than 20 EPEC effectors identified so far, four effectors have been reported to disrupt the intestinal barrier. These include EspF, Map, EspG1/G2 and NleA (Dean and Kenny, 2004; Glotfelty et al., 2014; McNamara et al., 2001; Philpott et al., 1996; Singh and Aijaz, 2016; Singh et al., 2018; Thanabalasuriar et al., 2010; Ugalde-Silva et al., 2016). Extensive studies to unravel the molecular basis of EPEC pathogenesis have been hampered by the absence of a suitable animal model that mimics human infection. In the absence of a human model, studies have mostly relied on a mouse model of infection as well as cell culture models (Law et al., 2013; Savkovic et al., 2005). These studies have highlighted the important roles of the EPEC effectors EspF and Map in mediating most of the disruptions in the host cell functions (Dean and Kenny, 2004; Dean et al., 2006; Holmes et al., 2010). For example, EspF inactivates the sodium hydrogen exchanger 3 (NHE3) and SGLT-1 while Map inactivates SGLT-1 and Na^+^/H^+^ exchanger regulatory factor-1/-2 (NHERF-1/-2) (Dean et al., 2006; Hodges et al., 2008; Martinez et al., 2010; Simpson et al., 2006). The paracellular space between the epithelial cells is sealed by the TJs, which selectively regulate the permeability of ions and solutes through this space (Farquhar and Palade, 1963; Krug et al., 2014; Zihni et al., 2016). The transmembrane proteins of the TJs such as claudins and occludin regulate the passage of ions and macromolecules across the paracellular space (Balda et al., 1996; Furuse et al., 1993, 1998; Krug et al., 2014; Zihni et al., 2016). Due to their apical localization, the TJs are frequently targeted by pathogens to disrupt the epithelial barrier (Croxen and Finlay, 2010). Both EspF and Map have been reported to cause extensive damage to the intestinal TJ barrier (Canil et al., 1993; Ma et al., 2006). Our earlier studies have shown that TJ disruption by EspF and Map is caused not only by the displacement of the TJ membrane proteins from the plasma membrane but also the significant depletion in their total amounts in the cells (Singh et al., 2018). We previously reported that the exogenous expression of EspF and Map caused a decrease in the transcripts of *occludin*, *claudin-1* and *claudin-4* and a decrease in the amounts of the corresponding proteins, possibly by post transcriptional regulation (Singh et al., 2018). We also showed that the depletion of TJ proteins was partially reversed in cells expressing EspF or Map after treatment with chloroquine, a lysosomal inhibitor (Singh et al., 2018). In continuation of that work, we further examined the involvement of the host cell lysosomes in the depletion of the TJ proteins by the EPEC effector, Map. Map is a 203 amino acid protein translocated into the infected cells via the T3SS (Dean and Kenny, 2009). Map harbors a mitochondrial targeting sequence (MTS) at the N-terminus between amino acid residues 1-44, a GTPase domain containing a conserved WxxxE motif between residues 74-78 and a PDZ Class1-binding domain containing the TRL motif at the C-terminus between amino acid residues 201-203 (Dean and Kenny, 2009). The N-terminal MTS of Map targets it to the mitochondria where it disrupts mitochondrial functions with the mitochondrial toxicity region (MTR) located between residues 101-152 playing an important role in altering the mitochondrial morphology (Papatheodorou et al., 2006). The GTPase domain allows Map to act as a guanine nucleotide exchange factor for the activation of Cdc42 at the plasma membrane, which regulates the formation of transient filopodia in infected cells during the early stages of infection (Huang et al., 2009). Activation of Cdc42 at the plasma membrane is mediated by the interaction between the TRL residues of Map and the PDZ domain of the ERM-binding phospho-protein 50 (Ebp50), also called NHERF-1, in an actin-dependent manner (Orchard et al., 2012; Simpson et al., 2006).

We first reported the involvement of the lysosomes in the EspF- and Map-mediated depletion of TJ proteins wherein we found that treatment of cells with chloroquine caused a significant increase in the protein levels of claudin-4 and occludin and to a lesser extent claudin-1 in cells expressing EspF and Map (Singh et al., 2018). This increase was more evident in cells expressing Map (Singh et al., 2018). Therefore, we further investigated the role of the lysosomal proteases in Map-mediated depletion of TJ proteins. Earlier studies have indicated that some TJ transmembrane proteins are targets of lysosomal cathepsins in different disease conditions. For example, the intestinal barrier breakdown in hemorrhagic shock is reported to be caused by the degradation of claudin-3 and occludin by the lysosomal cysteine protease, cathepsin B and the inhibition of cathepsin B prevented the degradation of these proteins (Klein et al., 2017). Inhibition of another lysosomal cysteine protease, cathepsin S, is reported to reverse TGF-β-induced epithelial–mesenchymal transition, restore the turnover of TJ proteins and prevent invasive growth in glioblastoma cells (Wei et al., 2021). In retinal pigment epithelial cells, increased exposure to recombinant cathepsin B disrupted the junctional localization of occludin and ZO-1 (Hadrian et al., 2019). Further, in mouse models of induced and spontaneous colitis, treatment with mannose prevented the intestinal barrier damage by inhibiting cathepsin B (Dong et al., 2022). To study the role of the host cell lysosomes in the Map-mediated depletion of TJ proteins, we treated cells expressing GFP-tagged Map with inhibitors of lysosomal proteases and assessed the effect of this treatment on the total amounts of the TJ proteins. We report here that the lysosomal cathepsins are involved in Map-mediated depletion of TJ membrane proteins. Further, by generating targeted deletions within Map, we have identified domains within Map that mediate the depletion of the TJ proteins as well as interactions with host proteins that regulate endocytosis and recycling such as caveolin-1 and Rab13, respectively.

## RESULTS

**Fig. 1.**
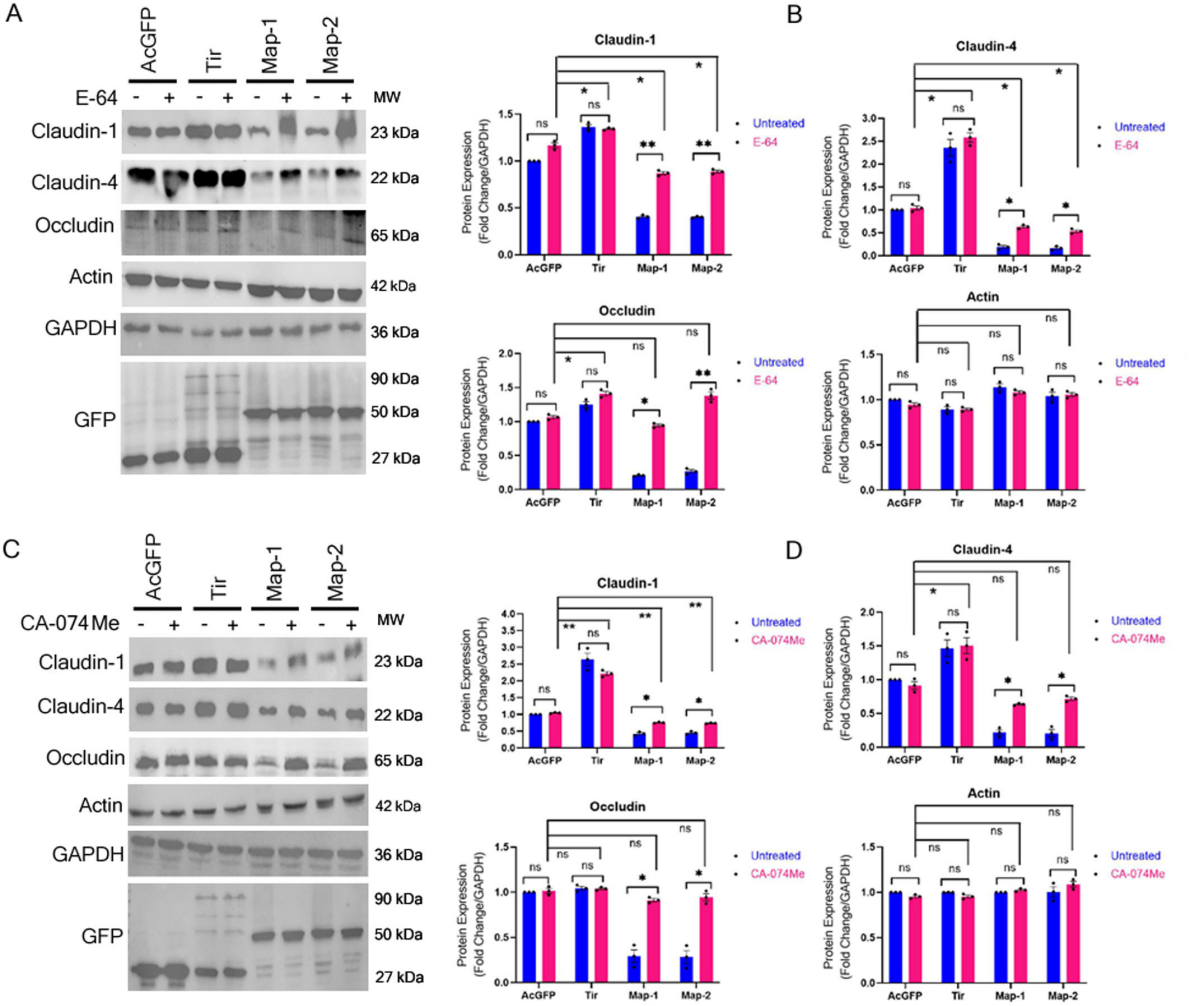
**Effect of E-64 and CA-074Me treatment on the expression of TJ proteins in cell lines expressing Map.** Stable cell lines expressing AcGFP vector (labeled AcGFP), AcGFP-Tir (labeled Tir) and two independent cell lines expressing AcGFP-Map (labeled Map-1 and Map-2) were treated with E-64 (A) and CA-074Me (C) for 18 h and cell lysates were prepared and subjected to western blotting with the indicated antibodies. GAPDH was used as a loading control. (B,D) The band intensities on the blots were analyzed by ImageJ software. Plots show fold changes in the expression levels of TJ proteins after treatment with E-64 or CA-074Me in cell lines expressing Map with respect to cell lines expressing AcGFP. Treatment with E-64 and CA-074Me increased the expression levels of claudin-1, claudin-4 and occludin in cell lines expressing GFP-Map but had no effect on actin levels. Data are represented as means±s.e.m. from at least three independent experiments. Horizontal lines in the chart represent statistical differences between groups. *P<0.05; **P≤0.008; ns, non significant.

To investigate whether the depletion of claudin-1, claudin-4 and occludin in cells expressing Map was mediated by the lysosomal cathepsins, we treated the cells with E-64, an irreversible inhibitor of cysteine proteases including cathepsin B, H, and L (Barrett et al., 1982) or CA-074 methyl ester (CA-074Me), a cell-permeable analog of CA-074, which irreversibly inhibits intracellular cathepsin B (Buttle et al., 1992). Stable cell lines expressing either the AcGFP vector alone or AcGFP-Map were grown in individual wells of a six-well plate until fully confluent and then treated with E-64 (30 µM) or CA-074Me (20 µM) for 18 h after which the total cell lysates were prepared and analyzed by western blotting (Fig. 1). Fold changes in the expression levels of TJ proteins in Map-expressing cells were calculated with respect to cells expressing the AcGFP vector (normalized to 1). Treatment of the two clonal cell lines expressing AcGFP-Map (Map-1 and Map-2) with E-64 increased the total protein levels of claudin-1 from ∼0.41 and ∼0.40 fold in untreated cells to ∼0.87 and ∼0.89 fold in treated cells. The total protein levels of claudin-4 increased from ∼0.19 and ∼0.16 fold in untreated cells to ∼0.63 and ∼0.53 fold in treated cells (Fig. 1A,B). The total protein levels of occludin changed significantly from ∼0.21 and ∼0.27 fold in untreated cells to ∼0.94 and ∼1.38 fold in E-64 treated cells (Fig. 1A,B). To show that this effect was specific for Map, we used cell lines expressing AcGFP-tagged translocated intimin receptor (Tir), another EPEC effector. Our earlier work has shown that cells exogenously expressing Tir displayed the same transepithelial resistance as wild-type MDCK cells and Tir expression did not displace claudins and occludin from the plasma membrane (Singh et al., 2018). However, Tir-expressing cells showed increased transcript numbers for *claudin-1*, *claudin-4* and *occludin* (Singh et al., 2018). Both untreated and E-64 treated cell lines expressing AcGFP-Tir exhibited average fold changes of ∼1.35 fold, ∼2.47 fold and ∼1.33 fold in the levels of claudin-1, claudin-4 and occludin, respectively with respect to E-64 treated cell lines expressing AcGFP vector alone (Fig. 1A,B). These data suggest that Tir stabilizes the expression of these TJ proteins. The mechanisms of Tir-mediated increase in the amounts of claudins and occludin is not clear and will be examined in future studies.

We also used the cell-permeable, intracellular cathepsin B inhibitor, CA-074Me. Treatment of the above cell lines with CA-074Me increased the total protein levels of claudin-1 from ∼0.42 and ∼0.45 fold in untreated cells to ∼0.76 and ∼0.74 fold in treated cells. The total amount of claudin-4 increased from ∼0.22 and ∼0.20 fold in untreated cells to ∼0.64 and ∼0.72 fold in treated cells. The total protein levels of occludin in Map-expressing cell lines increased from ∼0.30 and ∼0.28 fold in untreated cells to ∼0.91 and ∼0.94 fold in treated cells with respect to AcGFP cell lines treated with CA-074Me (Fig. 1C,D). The treatment of AcGFP-Map cell lines with E-64 or CA-074Me resulted in a significant increase in the protein levels of claudin-1, claudin-4 and occludin, which suggests that Map modulates cathepsin B to cause the depletion of claudin-1, claudin-4 and occludin in these cells. Treatment of cell lines expressing AcGFP-Tir with CA-074Me caused an increase in the protein levels of claudin-1 and claudin-4 by an average of ∼2.52 fold and ∼1.48 fold, respectively, in both treated and untreated cells. No significant change was seen in the levels of occludin in CA-074Me-treated AcGFP-Tir cells. In EPEC-infected cells, the Map-Ebp50-ezrin complex is recruited to the actin attachment sites at the plasma membrane where Map regulates the functions of F-actin (Orchard et al., 2012). Since actin contractility causes the disruption of TJs, we examined whether the levels of actin changed in cell lines expressing Map after treatment with the inhibitors. No significant change was observed in the levels of actin in any cell line after treatment with E-64 or CA-074Me (Fig. 1A-D).

**Fig. 2.**
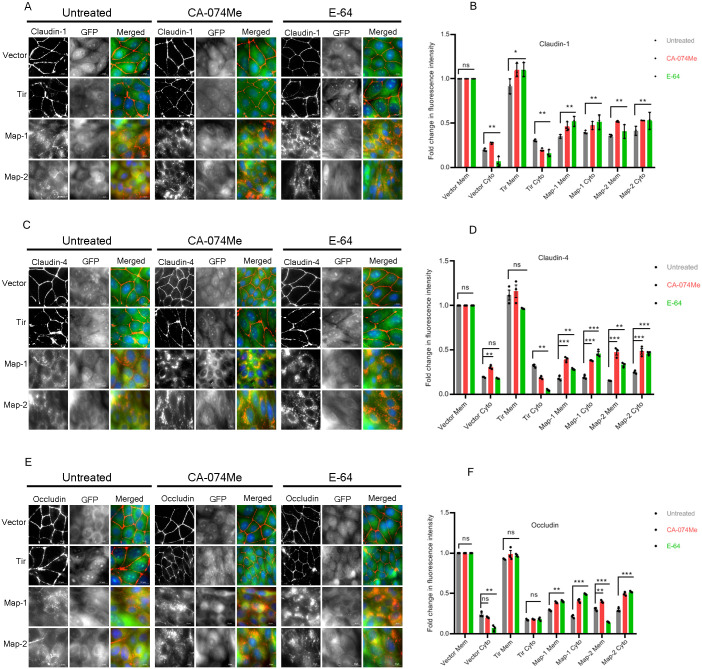
**E-64 and CA-074Me treatment of cell lines expressing GFP-Map increases the accumulation of TJ proteins in the cytoplasm.** Stable cell lines expressing AcGFP vector, GFP-Tir and GFP-Map (Map-1 and Map-2) were cultured on glass cover slips until they were 90% confluent and then treated with E-64 or CA-074Me as described in Materials and Methods. Untreated cells were kept as controls. The cells were fixed with chilled methanol and then labeled with antibodies against the TJ proteins claudin-1 (A), claudin-4 (C) and occludin (E). The localization of TJ proteins in these cell lines was examined by fluorescence microscopy. The intensity of each fluorescently labeled protein was quantitated by ImageJ software to measure the distribution of the TJ proteins at the plasma membrane and the cytoplasm. (B,D,F) Comparisons were made between cell lines expressing GFP-Map and cell lines expressing AcGFP vector. Increased accumulation of claudin-1, claudin-4 and occludin was observed in the cytoplasm of cells expressing GFP-Map but not in the control cell line expressing AcGFP vector or GFP-Tir where these proteins were localized at the TJs. TJ proteins: red; GFP-tagged proteins: green; nucleus: blue. Scale bar: 10 µm.

Next, we examined whether the increase in the protein levels of claudin-1, claudin-4 and occludin upon E-64 and CA-074Me treatment correlated with their increased localization at the plasma membrane. Cells expressing AcGFP vector alone, AcGFP-Tir or AcGFP-Map were grown on glass cover slips, treated with E-64 or CA-074Me and labeled with antibodies against claudin-1, claudin-4 and occludin. The cellular localization of these proteins was examined by immunofluorescence assays and the distribution of the TJ proteins was calculated by measuring the fluorescence intensity at the plasma membrane (normalized to1) and in the cytoplasm with respect to AcGFP-expressing cells by ImageJ analysis (Fig. 2). Treatment of the two Map-expressing cell lines with the inhibitors increased the localization of claudin-1 at the plasma membrane from ∼0.34 fold (Map-1) and ∼0.36 fold (Map-2) in untreated cells to ∼0.46 fold and ∼0.52 fold (Map-1), and ∼0.52 fold and ∼0.40 fold (Map-2) after treatment with CA-074Me and E-64, respectively. However, claudin-1 also exhibited increased localization in the cytoplasm with the fluorescence intensity increasing from ∼0.39 fold (Map-1) and ∼0.41 fold (Map-2) in untreated cells to ∼0.47 and ∼0.51 fold (Map-1) and ∼0.53 fold and ∼0.52 fold (Map-2) after treatment with CA-074Me and E-64, respectively (Fig. 2A,B). For claudin-4, the fluorescence intensity at the plasma membrane increased from ∼0.18 fold (Map-1) and ∼0.15 fold (Map-2) in untreated cells to ∼0.39 fold and ∼0.28 fold (Map-1) and ∼0.47 fold and ∼0.33 fold (Map-2) after treatment with CA-074Me and E-64, respectively. The cytoplasmic localization of claudin-4 increased from ∼0.19 fold (Map-1) and ∼0.24 fold (Map-2) in untreated cells to ∼0.38 fold and ∼0.46 fold (Map-1) and ∼0.48 fold and ∼0.46 fold (Map-2) in cells treated with CA-074Me and E-64, respectively (Fig. 2C,D). The fluorescence intensity of occludin at the plasma membrane changed from ∼0.29 fold (Map-1) and ∼0.31 fold (Map-2) in untreated cells to ∼0.39 fold and ∼0.40 fold (Map-1) and ∼0.40 fold and ∼0.15 fold (Map-2) in cells treated with CA-074Me and E-64, respectively. Occludin fluorescence intensity in the cytoplasm changed from ∼0.21 fold (Map-1) and ∼0.29 fold (Map-2) to ∼0.41 fold and ∼0.49 fold (Map-1), and ∼0.49 fold and ∼0.52 fold (Map-2) after treatment with CA-074Me and E-64, respectively (Fig. 2E,F). For all three membrane proteins, the fluorescence intensity in the cytoplasm significantly increased with respect to the cells expressing AcGFP vector after treatment with CA-074Me and E-64. These data suggest that treatment with these cathepsin inhibitors increases the localization of claudin-1/-4 and occludin in the cytoplasm rather than the plasma membrane. Even for occludin where the western blot data showed significant increase in total occludin levels, only discontinuous, patchy localization was seen at the TJs in cells treated with E-64 and CA-074Me, with most of the protein accumulating in the cytoplasm (Fig. 2E).

**Fig. 3.**
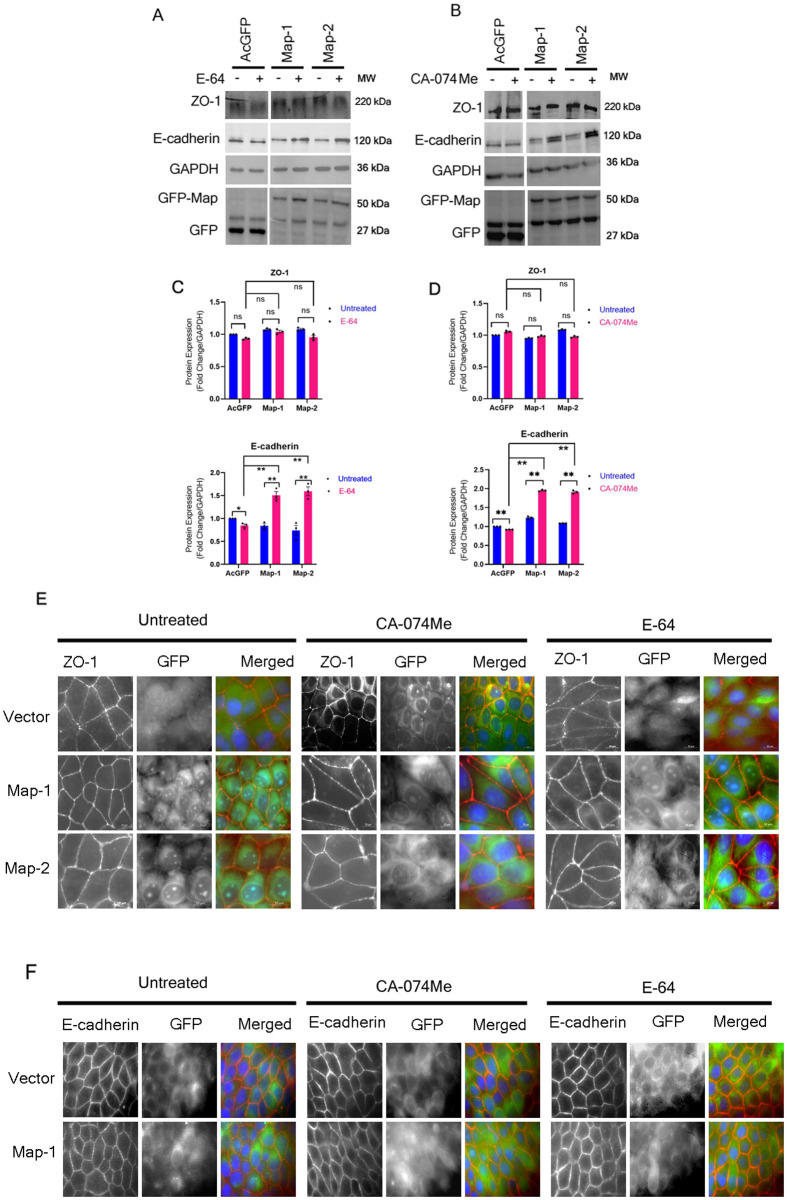
**Map does not affect the junctional localization of ZO-1 and E-cadherin.** Cell lysates derived from stable cell lines expressing AcGFP vector or Map (Map-1 and Map-2) were treated with E-64 or CA-074Me and the expression levels of ZO-1 and E-cadherin were examined (A-D). The expression levels of ZO-1 in untreated Map cell lines were similar to the levels in cell lines expressing AcGFP vector and treatment with E-64 or CA-074Me had no effect on ZO-1 expression in any cell line. However, the expression of E-cadherin increased after E-64 and CA-074Me treatment. *P<0.05; **P≤0.008; ns, non significant. No change was seen in the junctional localization of ZO-1 (E) and E-cadherin (F) in Map cell lines before or after treatment with E-64 or CA-074Me. Scale bar: 10 µm.

To rule out the possibility that E-64 and CA-074Me may have a general effect of increasing the expression of all TJ proteins, we examined the protein levels of the TJ adaptor protein ZO-1 in cell lines treated with these inhibitors (Fig. 3). No significant change in the amount of ZO-1 was observed between untreated and treated samples in each set. Further, the protein levels of ZO-1 in E-64 and CA-074Me treated cell lines expressing Map were similar to AcGFP cell lines (Fig. 3A-D). Additionally, ZO-1 displayed a normal localization at the TJs in all the cell lines (Fig. 3E). In epithelial cells, junction assembly starts with the recruitment of E-cadherin to the points of initial cell–cell contact followed by the recruitment of TJ proteins (Campbell et al., 2017). Therefore, we examined whether the reduction in the levels of the TJ membrane proteins in cell lines expressing Map was due to the reduced levels of E-cadherin at the plasma membrane. However, we observed that even untreated cell lines expressing Map did not show a reduction in E-cadherin levels compared with cell lines expressing AcGFP vector alone (Fig. 3A-D). However, in cells expressing Map, the protein levels of E-cadherin were found to increase to ∼1.5 fold (Map-1) and ∼1.6 fold (Map-2) after treatment with E-64 and ∼1.95 fold (Map-1) and ∼1.91 fold (Map-2) after treatment with CA-074Me (Fig. 3C-D). E-cadherin is reported to be degraded via the lysosomes (Cadwell et al., 2016; Palacios et al., 2005) so the increase in its protein levels is likely due to the inhibition of lysosomal proteases by E-64 and CA-074Me. Immunofluorescence assays showed a normal plasma membrane localization of E-cadherin in all the cell lines (Fig. 3F). These data indicate that E-64 and CA-074Me treatment specifically increases the protein levels of claudin-1, claudin-4 and occludin but not ZO-1 or E-cadherin in cell lines expressing Map.

**Fig. 4.**
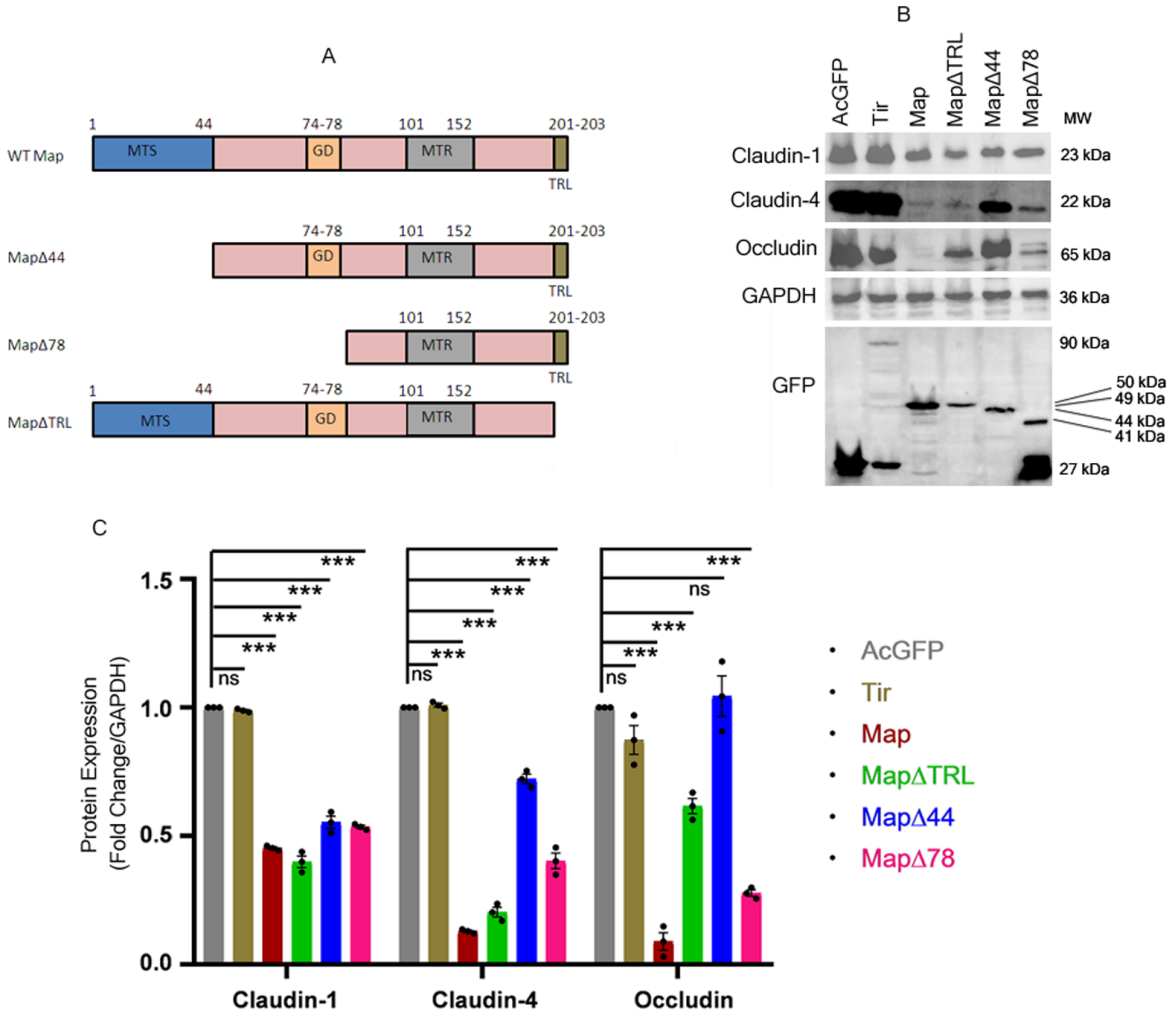
**Different Map domains mediate the depletion of TJ proteins.** (A) Schematic representation of Map showing the different domains. MTS: Mitochondrial targeting sequence; GD: GTPase domain containing WxxxE motif; MTR: Mitochondrial toxicity region; TRL: PDZ class 1-binding domain containing amino acid residues threonine, arginine and leucine; (B) Cell lysates derived from stable cell lines expressing AcGFP, GFP-Tir, GFP-Map, GFP-MapΔ44, GFP-MapΔ78 and GFP-MapΔTRL were analyzed by western blotting to identify domains within Map that mediate the depletion of TJ proteins. GAPDH was used as a loading control. (C) The band intensities were measured using ImageJ software. Fold changes in the expression of different TJ proteins in all Map cell lines were calculated with respect to cell lines expressing AcGFP vector (normalized to 1). Data are represented as means±s.e.m. from at least three independent experiments. ***P<0.0005; ns non significant.

**Fig. 5.**
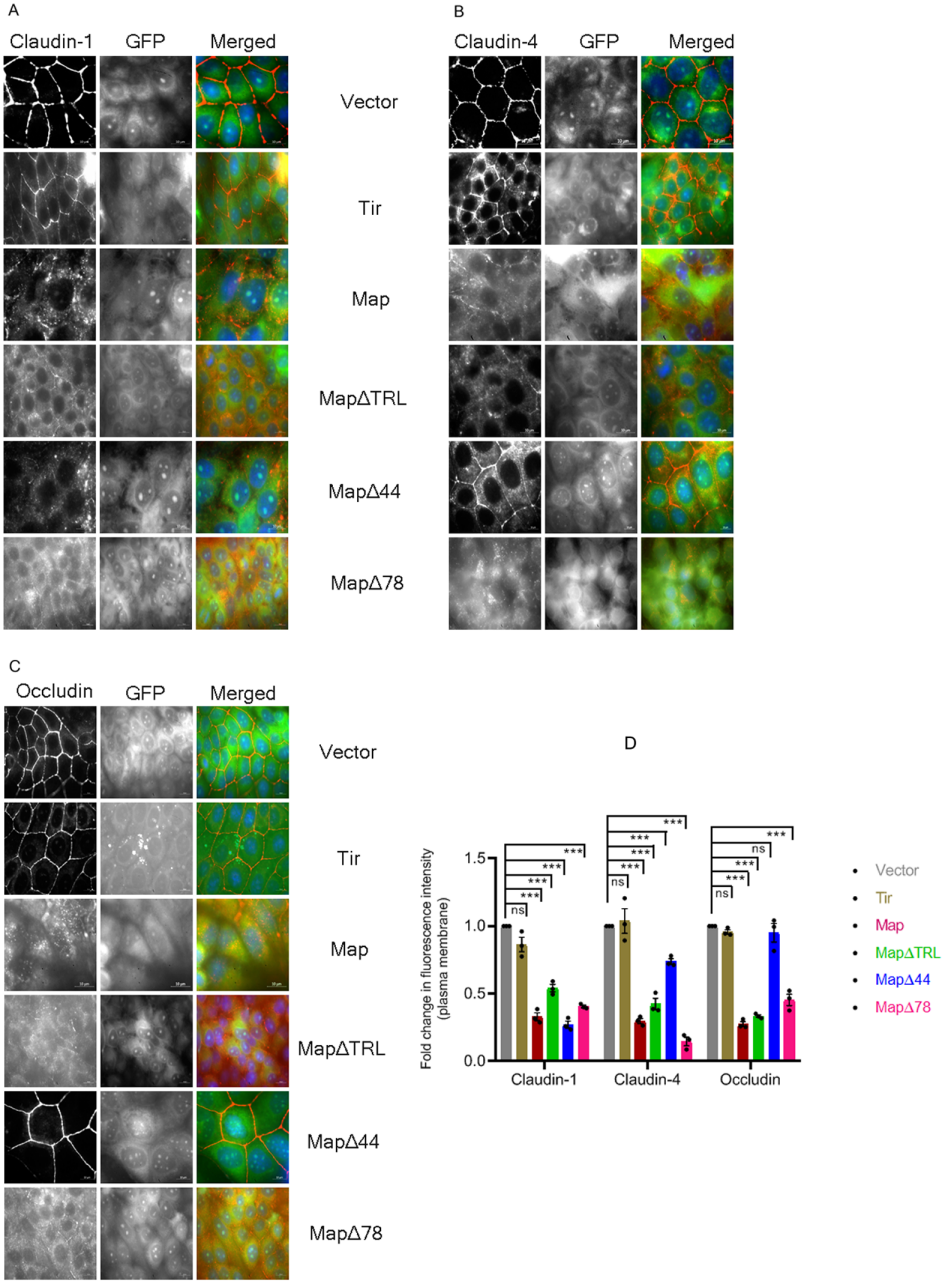
**Localization of TJ proteins in cells expressing different Map domains.** Stable cell lines expressing AcGFP vector, GFP-Tir, GFP-Map, GFP-MapΔ44, GFP-MapΔ78 or GFP-MapΔTRL were grown on cover slips, fixed with chilled methanol and labeled with antibodies against the TJ proteins claudin-1 (A), claudin-4 (B) and occludin (C) to examine their cellular localization. The fluorescence intensity of labeled proteins at the plasma membrane was quantitated by ImageJ analysis. (D) Plot shows relative fluorescence intensities of claudin-1, claudin-4 and occludin at the plasma membrane in Map cell lines with respect to AcGFP cell lines. Data are represented as means±s.e.m. from at least three independent experiments. ***P<0.0005; ns, non significant. TJ proteins: red; GFP-tagged proteins: green; nucleus: blue. Scale bar: 10 µm.

In order to identify domains within Map that may mediate the depletion of TJ proteins, we generated targeted N-terminal and C-terminal deletions within Map to generate mutant proteins that lacked the MTS located between amino acid residues 1-44 (MapΔ44), lacked the MTS and the GTPase domain containing the conserved WxxxE motif between residues 74-78 (MapΔ78), and lacked the TRL motif at the C-terminus between amino acid residues 201-203 (MapΔTRL). MDCK cell lines stably expressing these N-terminally GFP-tagged truncated proteins were generated and examined for their effect on the depletion of TJ proteins (Fig. 4A-C). Fold changes in the levels of TJ proteins in cell lines expressing different Map constructs were calculated with respect to cell lines expressing AcGFP vector. Cell lines expressing full length Map showed claudin-1 levels to be ∼0.45 fold while cell lines expressing MapΔTRL, MapΔ44 and MapΔ78 showed fold changes of ∼0.39, ∼0.55 and ∼0.54 fold, respectively as compared to cell lines expressing AcGFP vector alone. For claudin-4, a significant increase of ∼0.72 fold was seen in cell lines expressing MapΔ44 (Fig. 4B,C) while cells expressing full length Map, MapΔTRL and MapΔ78 exhibited claudin-4 levels of ∼0.13, ∼0.2 and ∼0.4 fold, respectively. Similarly, in cell lines expressing MapΔ44, the total levels of occludin were comparable to the control AcGFP cell line (Fig. 4B,C). Occludin levels changed to ∼0.6 fold in cells expressing MapΔTRL with respect to control cells expressing AcGFP vector. The levels of occludin in cells expressing full length Map and cells expressing MapΔ78 were ∼0.09 and ∼0.28 fold, respectively. These data suggest that the N-terminal MTS region of Map is involved in the depletion of occludin and claudin-4 as its removal (in MapΔ44 construct) increases the protein levels of occludin and claudin-4 significantly. As cells expressing MapΔTRL also exhibited increased levels of occludin, it is possible that the C-terminus of Map has an as-yet-unidentified role in the depletion of occludin. Intriguingly, the MapΔTRL construct contains both the MTS and the mitochondrial toxicity region, yet the amount of occludin in MapΔTRL cell lines was found to be significantly more (∼0.6 fold) than in cells expressing full length Map. Immunofluorescence data showed a complete junctional localization of occludin (∼0.95 fold) and partial junctional localization of claudin-4 (∼0.74 fold) in cells expressing MapΔ44 with respect to control cells expressing the AcGFP vector (Fig. 5B-D). However, cells expressing MapΔTRL showed only marginal increase (∼0.33 fold) in the localization of occludin at the TJs (Fig. 5C) even though the western blotting data showed a significant increase in the total levels of occludin in cells expressing MapΔTRL.

MapΔTRL-expressing cells exhibited a ∼0.54 fold increase in the junctional localization of claudin-1 with respect to AcGFP-expressing cells (Fig. 5A,D). Taken together, these data suggest that both the N- and C-termini of Map are involved in the localization of TJ proteins at the plasma membrane with the N-terminal region from (residues 1-78) specifically regulating occludin and claudin-4 localization.

**Fig. 6.**
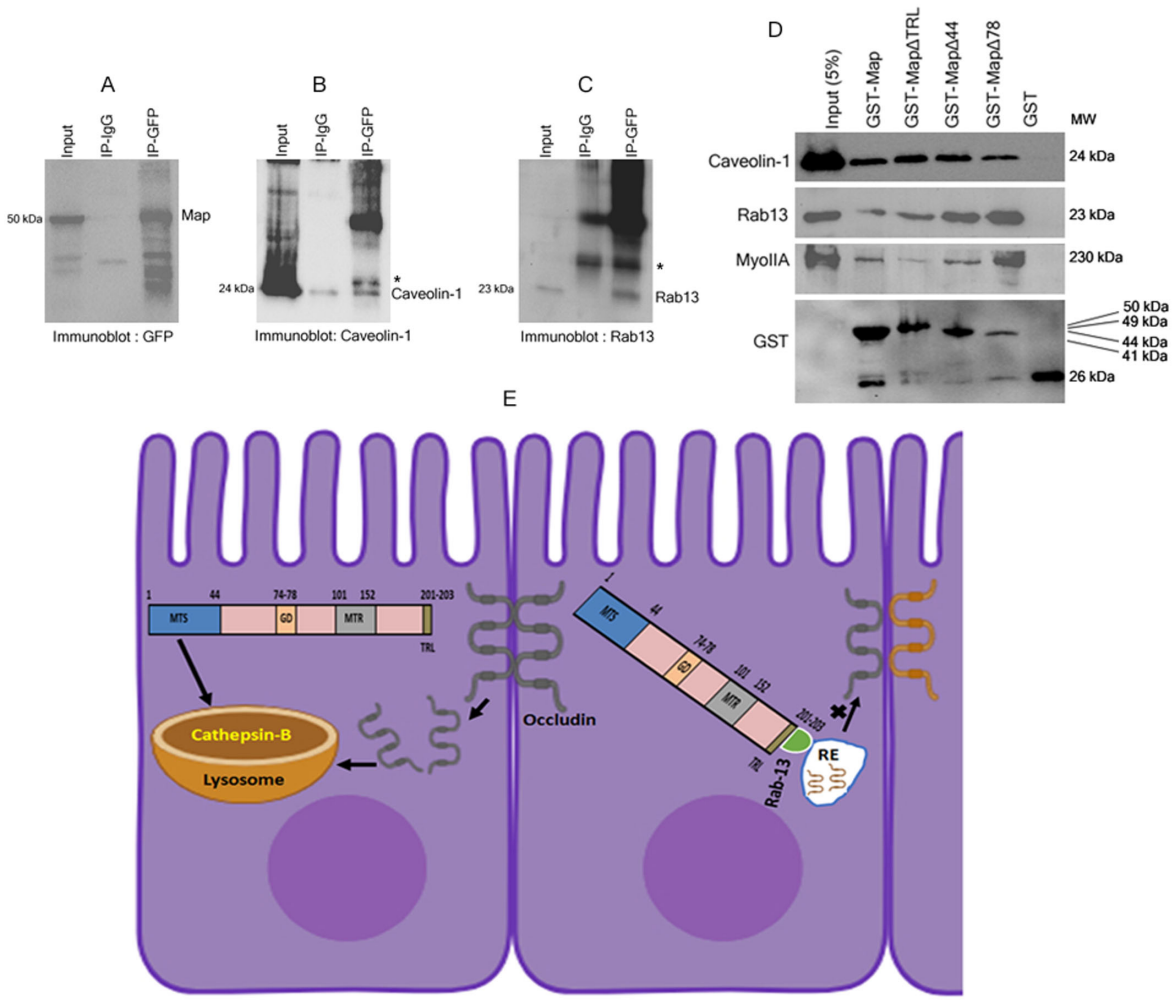
**Map interacts with host endocytosis proteins.** (A-C) Immunoprecipitation assays were performed with lysates derived from stable cell lines expressing full length Map and caveolin-1 and Rab13 were found to Co-IP with Map. Asterisk indicates IgG light chain. (D) Pull down assays were performed to identify the domains of Map that interact with caveolin-1, Rab13 and myosin IIA. (E) Schematic model showing the possible mechanisms of Map-mediated internalization and depletion of TJ proteins. RE: recycling endosomes.

Next, we examined whether the truncated Map proteins interacted with the host endocytosis machinery. Immunoprecipitation (IP) assays were carried out using anti-GFP antibody and the IP reactions were examined for the presence of markers of endocytosis (Fig. 6). Of the endocytosis markers examined, caveolin-1 and Rab13 were found to co-immunoprecipitate with Map (Fig. 6A-C). To identify the domains of Map that interact with caveolin-1 and Rab13, pull down assays were performed by immobilizing N-terminal GST-tagged Map proteins on glutathione sepharose beads and incubating them with MDCK cell lysates. Full length Map and all the truncated proteins were found to interact with caveolin-1 with similar efficiency (Fig. 6D) suggesting that Map likely internalizes the plasma membrane proteins via caveolin-mediated endocytosis. In addition, we found that the GTPase Rab13 strongly interacted with MapΔ44 and MapΔ78 proteins suggesting that the residues 1-78 of Map were not involved in the interaction with Rab13. Residues 1-44 of Map contain the mitochondrial targeting sequence while residues 74-78 contain the GTPase domain, which is involved in the activation of Cdc42 at the plasma membrane (Dean and Kenny, 2009; Huang et al., 2009). This suggests that the Map-mediated disruption of the mitochondria (via the MTS) may trigger the activation of endocytosis proteins at the plasma membrane. The efficiency of the Map-Rab13 interaction decreased when the TRL domain of Map was deleted (in MapΔTRL construct), which suggests that residues 79 to 203 likely mediate Rab13 interaction. We reported earlier that EPEC Map interacts with myosin IIA (Singh et al., 2018). Therefore, we identified the domain of Map that interacts with myosin IIA. Pull-down assays showed that MapΔ78 protein interacted strongly with myosin IIA while MapΔTRL protein showed a weak interaction (Fig. 6D). This indicates that the Map–Myosin IIA interaction likely occurs at the plasma membrane as the TRL domain is required for the transport of Map to the plasma membrane. Map is also reported to reorganize the cortical actin network in the sub-apical region for the activation of Cdc42 (Orchard et al., 2012) and this may be occurring through its interaction with myosin IIA. The TRL domain may serve to link the Map-Ebp50-ezrin-actin complex with myosin IIA to cause actin contractility and contribute to the leakage of solutes across the TJ barrier in EPEC infections as myosin IIA is an important regulator of actin contractility and opening of the TJ barrier (Zihni et al., 2016).

## DISCUSSION

We reported earlier that the EPEC effector Map depletes the levels of TJ membrane proteins and that the lysosomes may be involved in this process (Singh et al., 2018). To further understand the role of the lysosomes in the depletion of claudin-1, claudin-4 and occludin in stable cell lines expressing Map, we assessed the expression levels of these proteins after treatment with inhibitors of lysosomal cathepsins. Treatment with E-64 (a broad range inhibitor of cysteine proteases) and CA-074Me (a cell permeable inhibitor of cathepsin B) caused an increase in the expression levels of claudin-1, claudin-4 and occludin in cell lines expressing Map, suggesting that the Map-induced depletion of the TJ membrane proteins occurs via a cathepsin B-mediated pathway. However, the increase in the total levels of these TJ membrane proteins (observed in western blots) after the inhibition of cathepsin B did not result in their increased localization at the TJs and these proteins continued to be accumulated in the cytoplasm. The total protein levels and the junctional localization of the TJ cytoplasmic adaptor protein ZO-1 were not changed, nor was the plasma membrane localization of the adherens junction protein E-cadherin, which is reported to be degraded via the lysosomes (Cadwell et al., 2016; Palacios et al., 2005). Thus, Map specifically depletes the TJ transmembrane proteins claudin-1, claudin-4 and occludin. A recent study has reported that EPEC causes the secretion of cathepsin D and β-hexosaminidase into the extracellular space and the aberrant appearance of the lysosomal membrane protein Lamp1 on the plasma membrane of infected cells in an EspF/Map-dependent manner (Shtuhin-Rahav et al., 2023). Whether cathepsin B is also secreted into the extracellular media or whether Map activates cathepsin B or only accelerates the trafficking of the TJ membrane proteins to the lysosomes will be examined in future studies. Our study provides further evidence that Map modulates the lysosomes to deplete the TJ membrane proteins and consequently disrupt the TJ barrier.

We identified the domains of Map that may be involved in the depletion of TJ membrane proteins. Map mutant constructs lacking one or more domains were used to generate stable cell lines expressing these mutant Map proteins. Total cell lysates derived from these stable cell lines were analyzed by western blotting to check for changes in the protein levels of claudin-1/-4 and occludin. Lysates derived from cells expressing a truncated Map protein lacking the first 44 residues (MapΔ44) showed an increase in the expression of occludin comparable to that seen in control cell lines expressing the vector alone. Occludin expression also increased significantly in cells expressing the MapΔTRL construct as compared to cells expressing full length Map. The expression of claudin-4 significantly increased in cell lines expressing the MapΔ44 construct. These observations suggest that mitochondrial disruption, through the MTS of Map, mediates the depletion of occludin and claudin-4 and deletion of the MTS (in MapΔ44 construct) prevents the depletion of occludin and claudin-4. The localization of Map at the plasma membrane may be required for the depletion of junctional occludin. This is substantiated by the observation that Map proteins lacking the TRL residues (required for Map localization at the plasma membrane) exhibit increased expression of occludin as compared to full length Map protein. These data also suggest that Map may employ distinct strategies to deplete different TJ membrane proteins as the expression of claudin-1 did not increase as significantly as occludin and claudin-4 in lysates derived from cells expressing the MapΔ44 or MapΔTRL constructs. Immunofluorescence assays performed on cells expressing the MapΔ44 construct (lacking the MTS region) showed a complete localization of occludin and partial localization of claudin-4 at the TJs, further supporting the role of the MTS in their depletion. However, occludin did not localize at the TJs in cells expressing MapΔTRL. The TRL residues mediate the interaction of Map with the Ebp50-ezrin complex, which, in turn, interacts with actin leading to the recruitment of the Map-Ebp50-ezrin complex to the plasma membrane and the localized activation of Cdc42 at the plasma membrane (Orchard et al., 2012). Whether actin has some role in Map-dependent depletion of TJ membrane proteins or whether the recruitment of Map to the plasma membrane (via its TRL-dependent interaction with Ebp50) is required to initiate the endocytosis of TJ proteins will be examined in future studies. Intriguingly, cells expressing the MapΔ78 construct did not show an increase in the expression levels of occludin and claudin-4 even though the MTS domain is deleted in this construct also. The MapΔ78 construct also lacks the GTPase domain but contains the MTR and TRL domains. As reported recently, the secretion of cathepsin D and β-hexosaminidase by Map is mediated via the GTPase domain and the MTR regions of Map (Shtuhin-Rahav et al., 2023). Therefore, one reason that MapΔ78-expressing cells did not show an increase in the expression of claudin-4 and occludin could be that the GTPase domain somehow cooperates with the MTS and/or the MTR region to cause the increase in the expression of these TJ proteins.

As discussed above, the increase in the protein levels of claudin-1, claudin-4 and occludin after treatment with E-64 or CA-074Me did not result in the increased localization of these proteins at the TJs, suggesting that Map may inhibit their transport from the cytoplasm to the plasma membrane. Based on this data, we speculated that Map may modulate the endocytosis machinery to delocalize the existing TJ membrane proteins and prevent the recycling of the cytoplasmic pool of claudin-1, claudin-4 and occludin to the plasma membrane. To examine whether Map interacts with markers of endocytosis, we performed co-immunoprecipitation (Co-IP) and pull-down assays. Caveolin-1 and Rab13 were found to Co-IP with Map, indicating that Map may be modulating the endocytosis machinery. Pull-down assays revealed that truncated Map proteins lacking the first 44 or 78 residues or the C-terminal TRL residues were able to interact with caveolin-1 with the same efficiency. However, the interaction with Rab13 was most efficient when the first 78 residues of Map were deleted. The residues 1-44 of Map contain a MTS, suggesting that the interaction between Map and Rab13 does not depend on the targeting of Map to the mitochondria. This was further substantiated by increased efficiency of the Rab13 pull down by the truncated MapΔ44 protein as compared to full length Map protein. The Map-Rab13 interaction also does not require the GTPase activity of Map as the MapΔ78 protein does not contain the GTPase domain. The efficiency of the Rab13 pull down in the Map mutant protein lacking the TRL residues (MapΔTRL) was comparable to that seen with full length Map protein. The TRL residues are required for the plasma membrane recruitment of Map, so it is possible that Map may be interacting with Rab13 in the cytoplasm. As we had reported earlier that Myosin IIA Co-immunoprecipitates with Map (Singh et al., 2018), we examined the domains of Map that mediate this interaction. Truncated Map proteins lacking residues 1-78 also efficiently interacted with myosin IIA in pull-down assays. It has been reported that the activity of non-muscle myosin II is required for the trafficking of early endosomes and its inhibition disrupts the recycling of transferrin (Kar et al., 2023). Whether Map interacts with Rab13 at the same time as myosin IIA will be examined in future studies. Map proteins lacking residues TRL were not efficient in interacting with myosin IIA indicting that the membrane localization of Map is required for this interaction. Rab13 plays a role in the transport of proteins from the trans-Golgi network to the recycling endosomes and has been reported to regulate the recycling of occludin to the plasma membrane (Morimoto et al., 2005). EPEC is reported to modulate Rab5a and Rab11 in the regulation of protein trafficking and polarity in an EspF-dependent manner (Kassa et al., 2019; Tapia et al., 2017). The interaction of Map with Rab13 reported here adds to a growing list of GTPases that are targeted by EPEC to disrupt host cell functions by modulating the host endocytosis pathways.

In conclusion, our data suggest that Map may cause the internalization of TJ transmembrane proteins claudin-1, claudin-4 and occludin by modulating the host endocytosis pathways and the internalized proteins are depleted through mechanisms involving cathepsin B (Fig. 6E).

## MATERIALS AND METHODS

### Generation of plasmid constructs and recombinant Map proteins

The following primers containing restriction sites for EcoRI (at the 5′-end) and SalI (at the 3′-end) were used to generate full length and truncated *Map* constructs:

Map forward: 5′-AAAAAGAATTCCCTTAAGATGGTTAGTCCAACGGCAATGGTA-3′;

Map reverse: 5′-AAAAATCTAGAGTCGACCAGCCGAGTATCCTGCACATTGT-3′;

MapΔ44- forward: 5′-AAAAAGAATTCCCTTAAGATGTCGAACCTTATGATTAATC-3′;

MapΔ78- forward: 5′-AAAAAGAATTCCCTTAAGATGCAGATTACTTTTCTATCCA-3′;

MapΔTRL- reverse: 5′-AAAAATCTAGAGTCGACATCCTGCACATTGTCTGCA-3′.

PCR was performed by following standard protocols. Genomic DNA from Enteropathogenic *E. coli* O127:H6 strain E2348/69 was used as a template to generate *Map* constructs expressing full-length Map, MapΔTRL (lacking the C-terminal TRL residues), MapΔ44 (lacking residues 1 to 44) and MapΔ78 (lacking residues 1 to 78) proteins. The PCR products were cloned in pAcGFP1-C1 vector (for N-terminal GFP tag) or pGEX-4T-3 vector (for N-terminal GST tag) between the EcoRI and SalI sites of both vectors.

Additionally, GFP-tagged Tir construct was generated by using primers containing restriction sites for BamHI (at 5′-end) and SalI (at 3′-end): Tir forward: 5′-AAAAAGGATCCCTTAAGATGGCTATTGGTAACCTTGGT-3′;

Tir reverse: 5′-AAAAATCTAGAGTCGACAACGAAACGTACTGGTCCCGG-3′.

The resulting PCR products were cloned in pAcGFP1-C1 vector between the BglII (due to the generation of compatible BamHI overhangs) and SalI sites. Tir was used as a control EPEC effector as it does not deplete TJ proteins (Singh et al., 2018).

Recombinant GST-tagged Map proteins were obtained by transforming BL21(DE3)pLysS cells with the plasmid constructs cloned in pGEX-4T-3 vector. Cultures were induced with 1 mM IPTG and incubated overnight at 25°C to obtain GST, full-length GST-Map, GST-MapΔ44, GST-MapΔ78 and GST-MapΔTRL proteins. All recombinant constructs were generated after the approval of the institutional biosafety committee of Jawaharlal Nehru University.

### Generation of stable cell lines

MDCK cells, maintained in Dulbecco's Modified Eagle Medium (DMEM) supplemented with 10% Fetal Bovine Serum (FBS), were transfected with AcGFP1-C1 vector or the Map and Tir constructs cloned in pAcGFP1-C1 vector using Lipofectamine 3000 reagent (Thermo Fisher Scientific). Cells expressing AcGFP alone, full length Map, full length Tir and truncated Map proteins were selected by adding G418 (500 µg/ml) to the medium and incubated in a CO_2_ incubator at 37°C for 2-3 weeks. Subsequently, clones expressing AcGFP, GFP-Map, GFP-MapΔ44, GFP-MapΔ78, GFP-MapΔTRL and full length GFP-Tir were picked and propagated further. At least 10 clones were picked and analyzed for each cell line. Expression of all proteins was confirmed by western blotting with anti-GFP antibodies as well as by fluorescence microscopy. For each experiment, at least two independent clones (biological replicates) of Map were used. All cell lines were periodically tested for contamination.

### Immunofluorescence assays and microscopy

The cells expressing AcGFP vector alone, GFP-Tir, GFP-Map, GFP-MapΔ44, GFP-MapΔ78 and GFP-MapΔTRL were grown on glass cover slips until 90% confluent and then fixed with chilled methanol for 5 min followed by incubation in PBS for 5 min at room temperature. The cells were then incubated in blocking solution (containing 0.5% w/v BSA in PBS) for 30 min at room temperature followed by incubation with primary antibodies diluted 1:300 in solution containing 1× PBS, 0.5% BSA and 0.1% sodium azide for 2-4 h at room temperature. The cover slips were then rinsed thrice with PBS, 0.5% BSA and 0.1% sodium azide. The cells on cover slips were then incubated with Cy3-conjugated anti-mouse/anti-rabbit secondary antibodies (Millipore) for 1 h at room temperature. The cells were rinsed thrice and mounted in PBS, 0.5% BSA and 30% glycerol on glass slides. Images were obtained at 100× magnification on an ApoTome (Axiovert40 CFL, Zeiss). Primary antibodies used for labeling were claudin-1 (#374900, mouse monoclonal), claudin-4 (#329400, mouse monoclonal), occludin (#331500, mouse monoclonal), ZO-1 (#339100, mouse monoclonal) and E-cadherin (#717100, rabbit polyclonal pan cadherin), all purchased from Thermo Fisher Scientific and used at 1:300 dilution. For labeling the nucleus, DAPI (4′,6-diamidino-2-phenylindole (Millipore) was used at 1:5000 dilution.

### Preparation of total cell lysates

The total protein lysates were prepared from confluent cell lines expressing AcGFP vector alone, GFP-Map, GFP-MapΔ44, GFP-MapΔ78, GFP-MapΔTRL and GFP-Tir, grown on six-well plates. The total cell lysates were prepared by adding 1× Laemmli buffer to the wells of culture plates followed by extraction through a 23-gauge needle several times. The lysates were analyzed by electrophoresis on 12% SDS polyacrylamide gels followed by western blotting with primary antibodies at 1:1000 dilutions in blocking solution. The primary antibodies used were claudin-1, claudin-4, occludin, ZO-1, E-cadherin (described above) and GFP (#A01388, rabbit polyclonal, GenScript), all used at 1:1000 dilution. GAPDH (#AB0060, rabbit polyclonal, Biobharti Life Science; 1:10,000 dilution) was used as a loading control. Secondary antibodies used were HRP-conjugated anti-mouse or anti-rabbit antibodies (Millipore). After western blotting, the band intensities were analyzed by ImageJ software (https://imagej.net/ij) to obtain fold changes with respect to lysates derived from cell lines expressing AcGFP vector (normalized to 1). Uncropped blots are shown in Fig. S1.

### Treatment with E-64 and CA-074Me

For CA-074Me and E-64 treatment, equal number of cells from cell lines expressing AcGFP vector, full-length GFP-Map (clones Map-1 and Map-2) and GFP-Tir were seeded on a six-well plate. At 90% confluency, the cells were treated with either E-64 (30 µM) or CA-074Me (20 µM) for 18 h at 37°C and then rinsed with PBS. Total cell lysates, after treatment with E-64 or CA-074Me, were prepared as described above and analyzed by western blotting with the antibodies listed above. ImageJ was used to measure band intensities on blots and the relative fold changes with respect to cells expressing the vector were plotted on graphs.

Immunofluorescence assays were performed to assess changes in the localization of claudin-1, claudin-4 and occludin at the plasma membrane after treatment with E-64 or CA-074Me with respect to untreated cells. Confluent cells expressing the vector, GFP-Tir and GFP-Map (Map-1 and Map-2 clones), grown on cover slips, were treated with E-64 or CA-074Me as described above and labeled with antibodies against claudin-1, claudin-4 and occludin. The fluorescence intensity at the plasma membrane and the cytoplasm in the three groups of cells (i.e. untreated, E-64 treated and CA-074Me treated) was quantitated by ImageJ analysis. Measurements were taken from at least three cells from each cover slip. For each cell, three regions of interest (ROIs) at the plasma membrane and the cytoplasm were selected and the fluorescence was measured. The value obtained by measuring an area with no fluorescence was set as background and this background value was subtracted from the measurements taken for the plasma membrane and the cytoplasm. For each group, relative fluorescence intensities (fold change) at the plasma membrane in cells expressing GFP-Tir and GFP-Map (Map-1 and Map-2) were calculated with respect to the fluorescence intensity at the plasma membrane in cells expressing the vector (normalized to 1) and graphs were plotted. The fluorescence intensities in the cytoplasm of cells expressing GFP-Tir and GFP-Map were calculated with respect to the fluorescence intensity at the plasma membrane in cells expressing the vector in each group.

### Pull-down assays

Lysates derived from BL21(DE3)pLysS cells expressing GST, full-length GST-Map, GST-MapΔ44, GST-MapΔ78 and GST-MapΔTRL proteins were clarified by centrifugation and incubated with glutathione sepharose beads overnight at 4°C with constant rotation. The glutathione sepharose beads bound to GST, GST-Map, GST-MapΔ44, GST-MapΔ78 and GST-MapΔTRL were centrifuged, washed with lysis buffer containing 1× PBS, 1 mM DTT, 1 mM PMSF and 0.5% Triton X-100 three times and then incubated with lysates derived from wild-type MDCK cells for 12-14 h at 4°C on a rotary shaker. MDCK cell lysates were prepared by growing the cells on 100 mm culture plates until fully confluent after which 1 ml of pull-down buffer (1× PBS, 1 mM DTT, 1 mM PMSF and 0.5% Triton X-100) was added. Subsequently, the cells were harvested by scraping from the culture plates and passed through a 21-gauge needle several times and then incubated on ice for 30 min. The lysates were centrifuged, and the supernatant was incubated with glutathione sepharose beads bound to the GST proteins mentioned above overnight at 4°C with rotation. The beads were centrifuged, washed three times with cell lysis buffer, mixed with equal volumes of 2× SDS gel loading dye, resolved on 12% SDS polyacrylamide gels and analyzed by western blotting. The primary antibodies used were caveolin-1 (#3267S, Rabbit monoclonal, Thermo Fisher Scientific), Rab13 (#PA5-52039, Rabbit monoclonal, Thermo Fisher Scientific), MyosinIIA (#3403, rabbit polyclonal, Cell Signaling Technology) and GST (#GE27-4577-01, goat polyclonal, Sigma Aldrich; used at 1:10,000 dilution). HRP-conjugated anti-goat (#A5420) and anti-rabbit (#A6154) secondary antibodies (Merck) were used at 1:10,000 dilution.

### Immunoprecipitation assays

Confluent monolayers of cells stably expressing AcGFP-Map, grown in 100 mm cell culture plates, were washed with cold PBS and suspended in 2.5 ml of RIPA buffer (1% NP-40, 1% triton X-100, 0.5% sodium deoxycholate, 0.2% SDS, 150 mM NaCl, 20 mM HEPES, 2 mM EDTA, 0.2 mM PMSF). Cells were lysed by passing the cell suspension through a 21 gauge needle several times followed by incubation on ice for 30 min. The cell extract was then centrifuged at 12,000 rpm, for 15 min at 4°C and 750 µl of the supernatant was transferred to fresh tubes followed by the addition of either 1.5 µg of anti-GFP antibody (raised in rabbit) or 1.5 µg of Rabbit IgG. The samples were mixed overnight at 4°C by gentle rotation. Subsequently, 80 µl of protein G agarose beads (G-Biosciences, India) were added to each tube and the tubes incubated for 4 h at 4°C. The beads were washed several times with RIPA buffer and examined by western blot analysis.

### Statistical analysis

All experiments were performed at least three times. For each experiment two independent clones (biological replicates) expressing full length AcGFP-Map or truncated Map proteins were used. Data obtained from cell lines expressing full length Map or truncated Map proteins were compared with respect to cell lines expressing AcGFP vector and fold changes were calculated. Data were represented as means±s.e.m. from at least three independent experiments. Unpaired *t*-tests were used to calculate differences between these groups. Differences between two groups were considered significant at *P*-values <0.05.

## Supplementary Material

10.1242/biolopen.061794_sup1 Supplementary information
