# Supplementary material for: Enteropathogenic E. coli effector Map interacts with Rab13 and regulates the depletion of the tight junction proteins occludin and claudins via cathepsin B-mediated mechanisms
Source: Biol Open. 2025 Feb 27;14(2):BIO061794. doi: 10.1242/bio.061794 (PMC11892358; doi:10.1242/bio.061794)
Supplement: Supplementary information [file biolopen-14-061794-s1.pdf]

# Figure 1 A

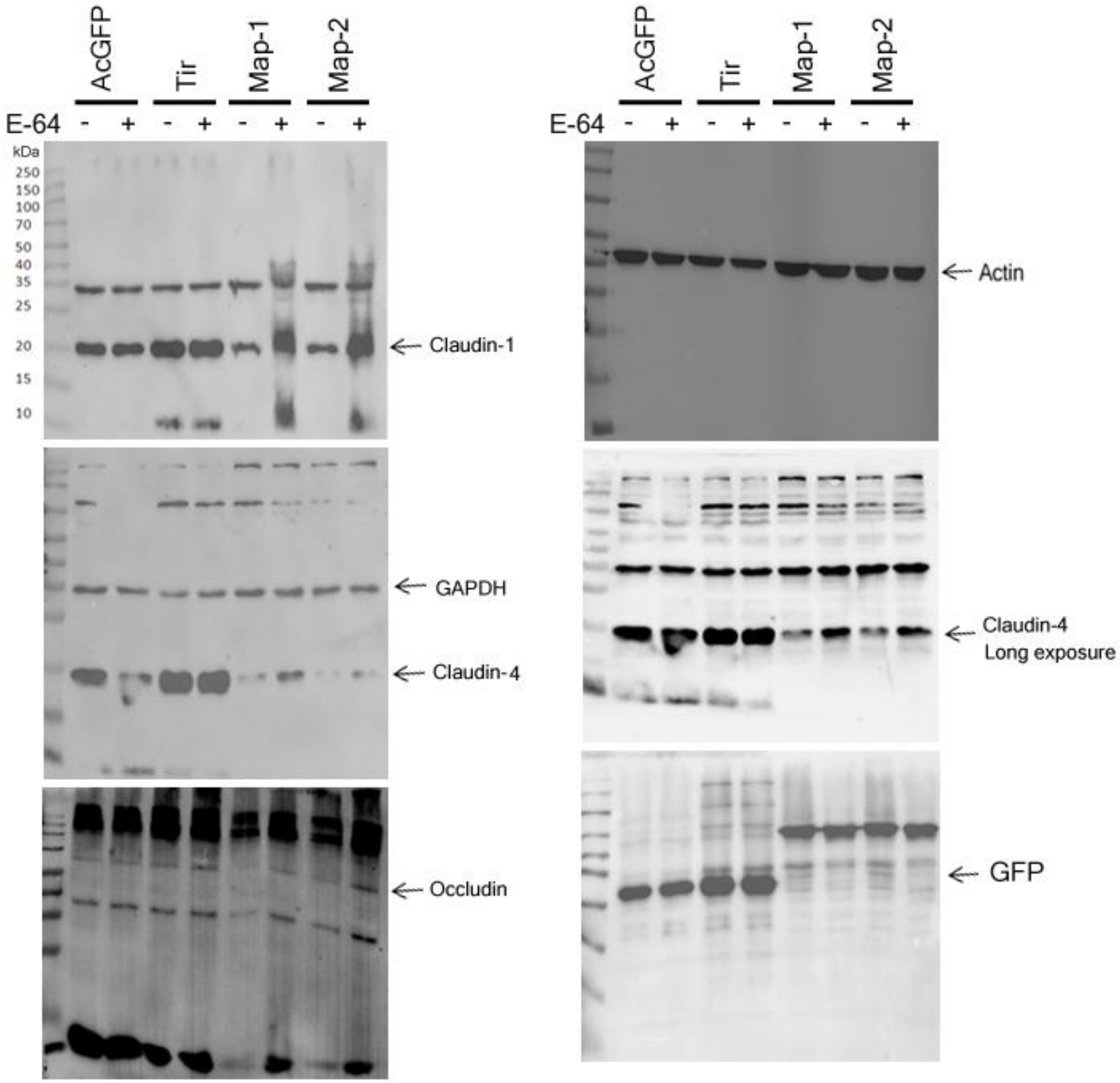


Biology Open • Supplementary information

**Figure 1 C**

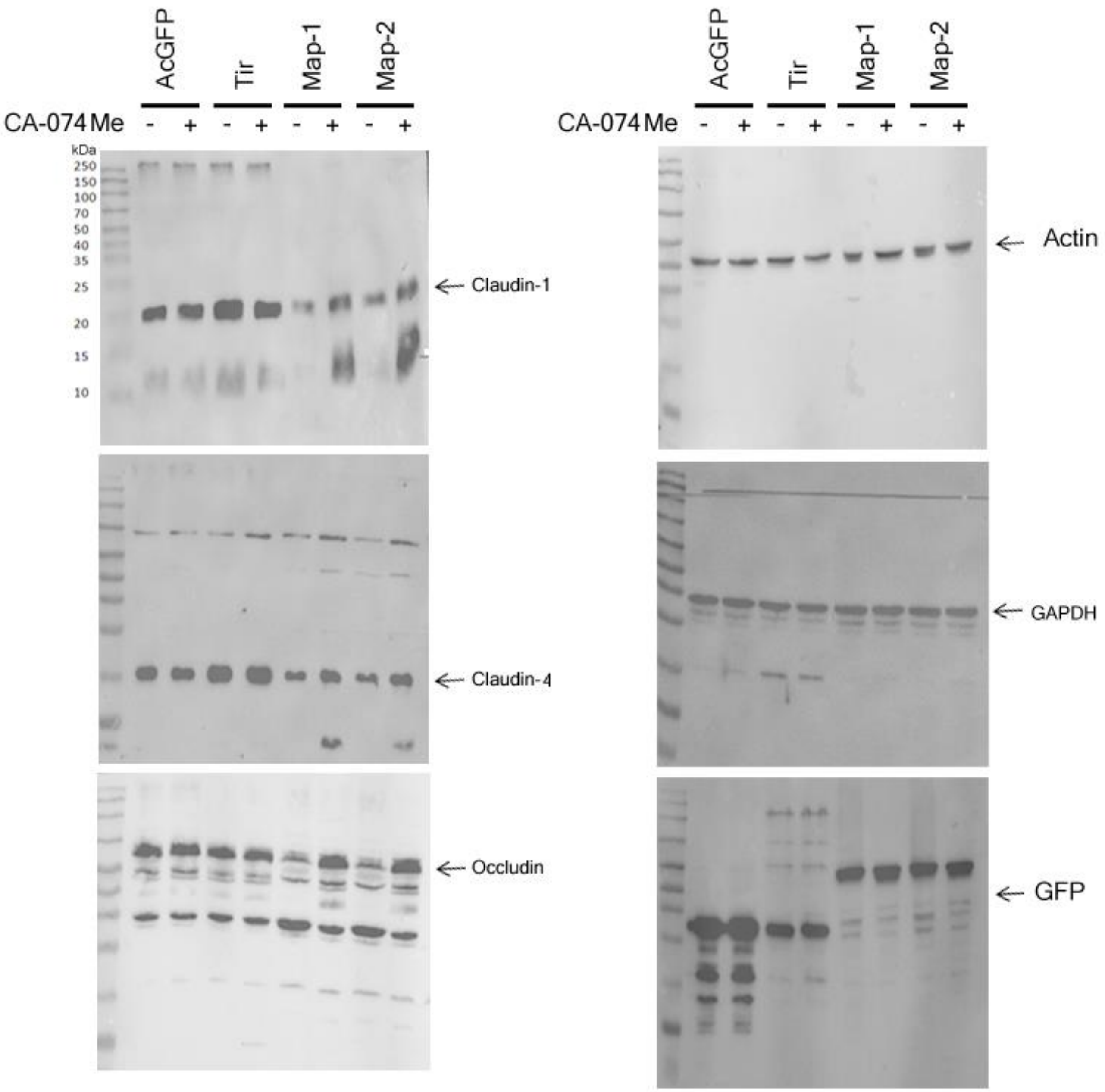


Biology Open • Supplementary information

**Figure 3 A**

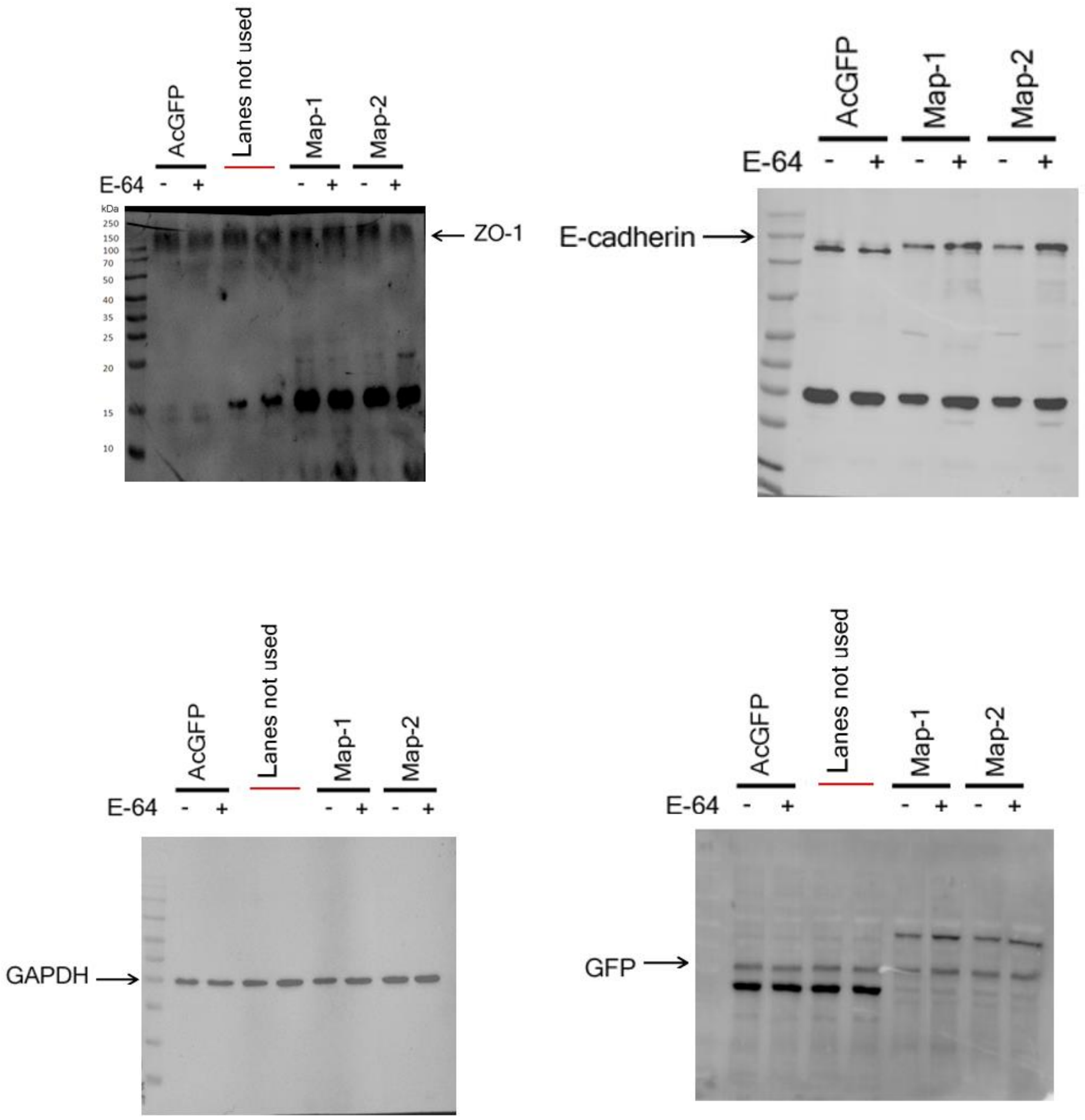
AcGFP
Lanes not used
Map-1
Map-2
E-64 - + - + - +
kDa
250
150
100
70
50
40
35
25
20
15
10
ZO-1
AcGFP
Map-1
Map-2
E-64 - + - + - +
E-cadherin
AcGFP
Lanes not used
Map-1
Map-2
E-64 - + - + - +
GAPDH
AcGFP
Lanes not used
Map-1
Map-2
E-64 - + - + - +
GFP

Biology Open (2025): doi:10.1242/bio.061794: Supplementary information

**Figure 3 B**

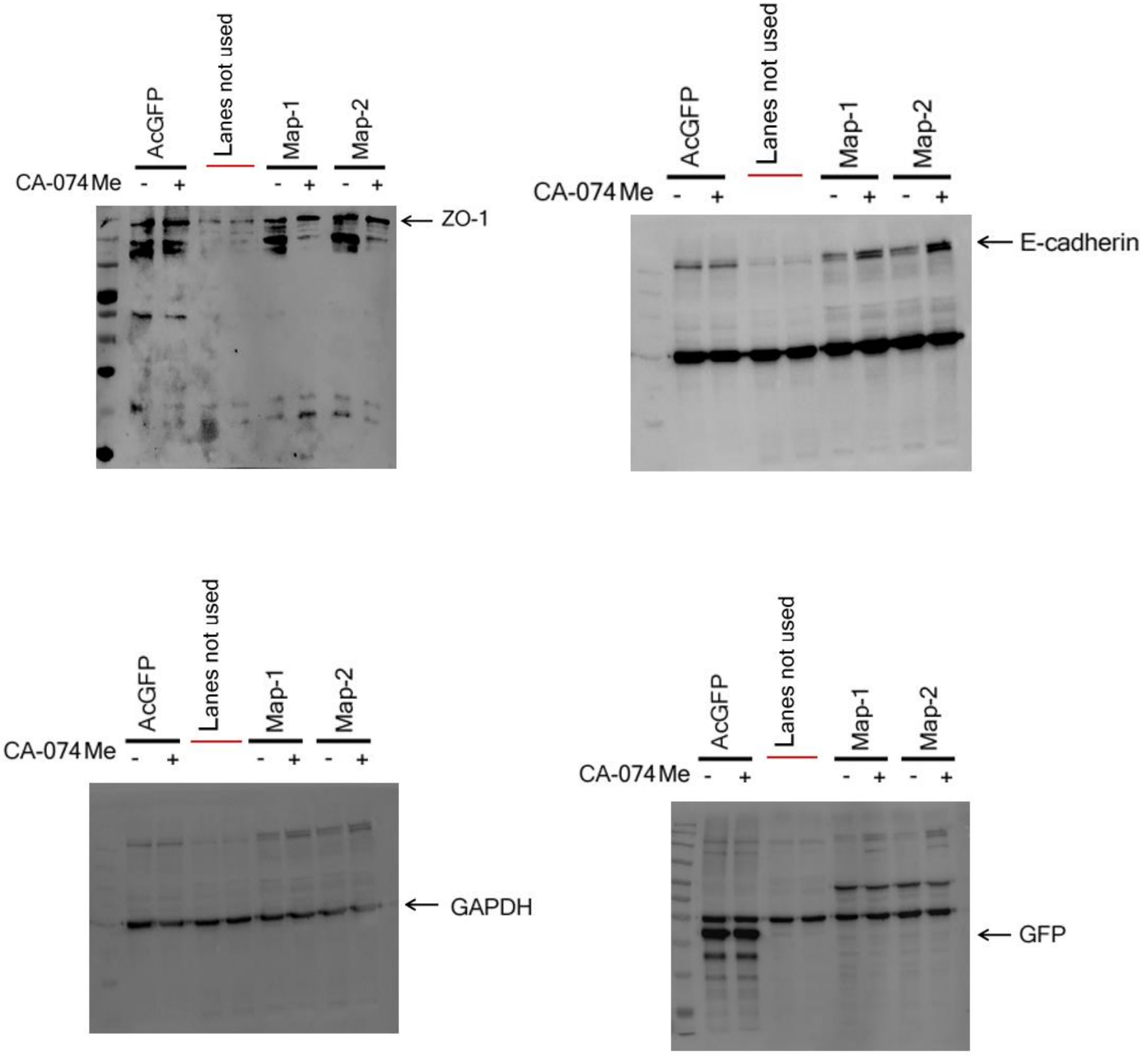


Biology Open • Supplementary information

**Figure 4B**

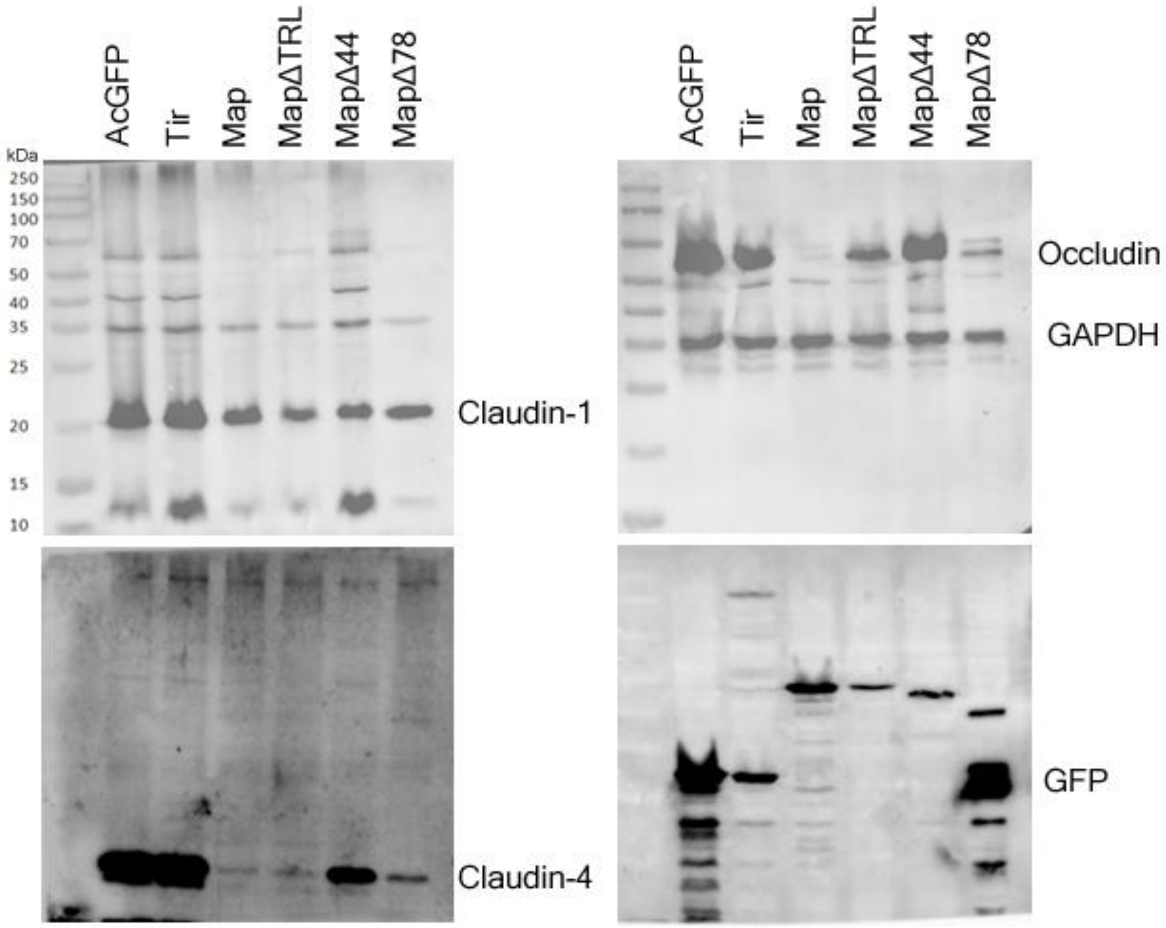


Biology Open • Supplementary information

# Figure 6

## Figure 6A

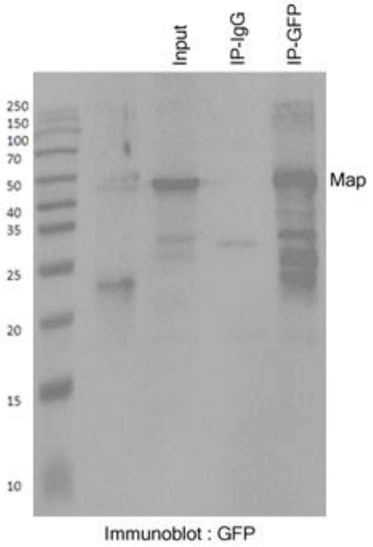


## Figure 6B

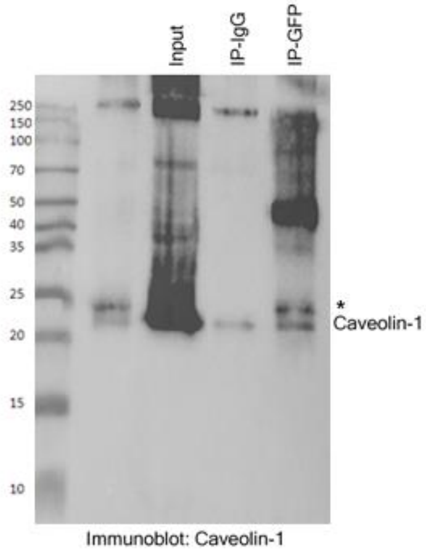


## Figure 6C

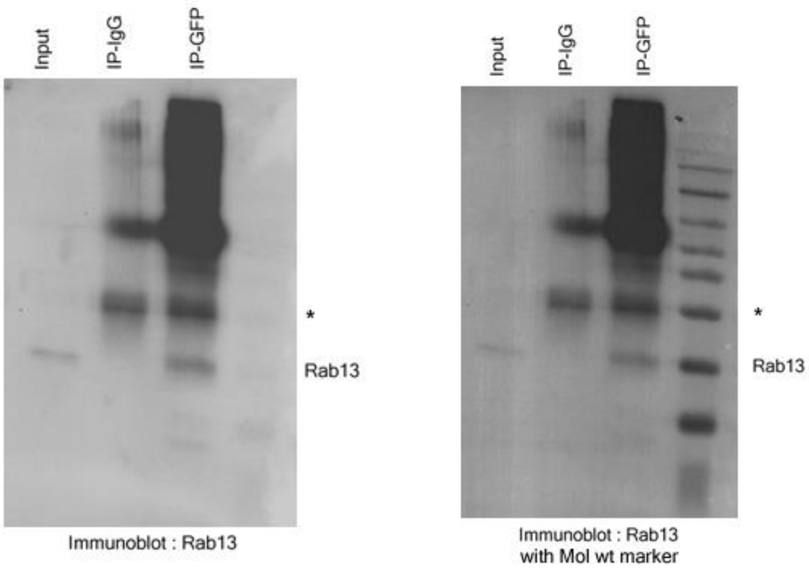


Biology Open • Supplementary information

# Figure 6 D

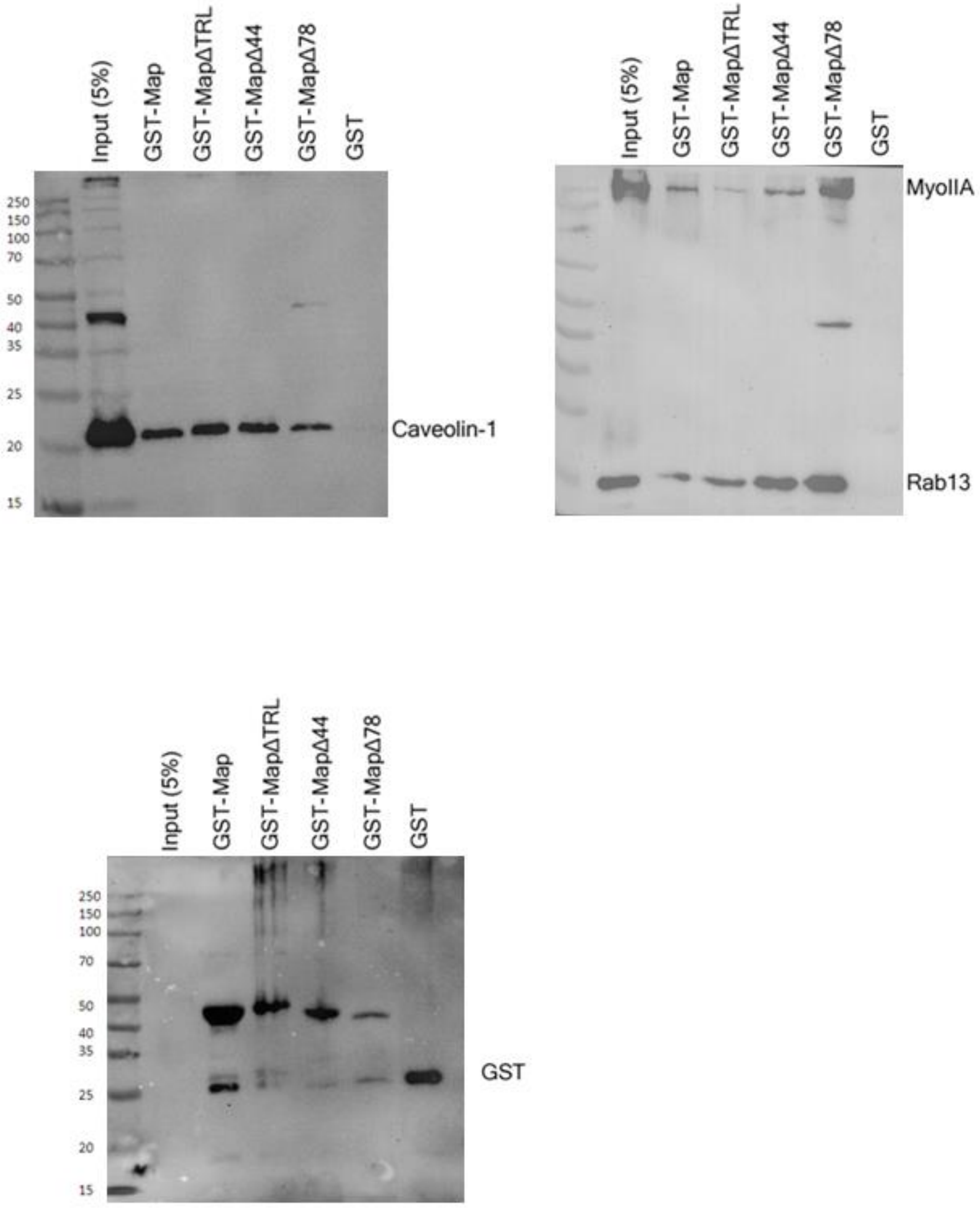


**Fig. S1. Uncropped Blots**
